# Supplementary material for: Effects of pesticide application on soil bacteria community structure in a cabbage-based agroecosystem in Ghana
Source: PLoS One. 2025 May 29;20(5):e0323936. doi: 10.1371/journal.pone.0323936 (PMC12121791; doi:10.1371/journal.pone.0323936)
Supplement: S1 Table — (DOCX) [file pone.0323936.s001.docx]

**SUPPLEENTARY DATA**

**S1 Table: Taxonomic hierarchy of bacteria species within the non-contaminated (NCS), abandoned pesticide-contaminated (AB-PCS) and active pesticide-contaminated (AC-PCS) soils.**

|  | **Treatment** | | | | | |
| --- | --- | --- | --- | --- | --- | --- |
| **Species** | **NCS** | **Percentage** | **AB-PCS** | **Percentage** | **AC-PCS** | **Percentage** |
| Unknown | 7357 | 71.03 | 3614 | 85.58 | 1,223 | 74.39 |
| *Bacillus* sp. | 540 | 5.21 | 18 | 0.43 | 12 | 0.73 |
| *Pseudomonas veronii* | 645 | 6.23 | 2 | 0.05 | - | - |
| *Prevotella albensis* | 371 | 3.58 | 2 | 0.05 | - | - |
| *Bacillus flexus* | 205 | 1.98 | 2 | 0.05 | 6 | 0.36 |
| *Prevotella ruminicola* | 184 | 1.78 | 16 | 0.38 | 8 | 0.49 |
| *Pseudomonas carboxydohydrogena* | 16 | 0.15 | 20 | 0.47 | 14 | 0.85 |
| *Rhodoplanes elegans* | 88 | 0.85 | 64 | 1.52 | 83 | 5.05 |
| *Butyrivibrio* | 69 | 0.67 | 2 | 0.05 | 6 | 0.36 |
| *Gemmata obscuriglobus* | 33 | 0.32 | 38 | 0.90 | 35 | 2.13 |
| *Nostocoida limicola* | 55 | 0.53 | 48 | 1.14 | 25 | 1.52 |
| *Nitrospira calida* | 9 | 0.09 | 28 | 0.66 | 35 | 2.13 |
| *Gemmata eligans* | 32 | 0.31 | 16 | 0.38 | 12 | 0.73 |
| *Solirubrobacter* sp. | 39 | 0.38 | 36 | 0.85 | 19 | 1.16 |
| *Cystobacter* sp. | 5 | 0.05 | 24 | 0.57 | 2 | 0.12 |
| *Singulisphaera rosea* | 20 | 0.19 | 10 | 0.24 | 10 | 0.61 |
| *Pedomicrobium australicum* | 12 | 0.12 | 26 | 0.62 | 9 | 0.55 |
| *Nitrospira* sp. | 18 | 0.17 | 18 | 0.43 | 15 | 0.91 |
| *Pedomicrobium* sp. | 68 | 0.66 | 2 | 0.05 | - | - |
| *Selenomonas lacticifex* | 65 | 0.63 | 2 | 0.05 | 6 | 0.36 |
| *Bacillus koreensis* | 29 | 0.28 | 2 | 0.05 | 1 | 0.06 |
| *Arthrobacter woluwensis* | 28 | 0.27 | 2 | 0.05 | 6 | 0.36 |
| *Alicyclobacillus* | 26 | 0.25 | 2 | 0.05 | 1 | 0.06 |
| *Paenibacillus curdianolyticus* | 36 | 0.35 | 6 | 0.14 | - | - |
| *Pseudomonas umsongensis* | 18 | 0.17 | 8 | 0.19 | - | - |
| *Clostridium butyricum* | 22 | 0.21 | 2 | 0.05 | - | - |
| *Eggerthella* sp. | 21 | 0.20 | 1 | 0.02 | - | - |
| *Candidatus versatilis* | 19 | 0.18 | 10 | 0.24 | 3 | 0.18 |
| *Treponema azotonutricium* | 20 | 0.19 | 4 | 0.09 | - | - |
| *Acidobacterium* sp. | 5 | 0.05 | 4 | 0.09 | 2 | 0.12 |
| *Bacteroides nordii* | 7 | 0.07 | - | - | 2 | 0.12 |
| *Rubrivivax* sp. | 2 | 0.02 | 6 | 0.14 | 2 | 0.12 |
| *Paenibacillus chondroitinus* | 10 | 0.10 | - | - | 2 | 0.12 |
| *Pirellula* | 7 | 0.07 | 6 | 0.14 | 2 | 0.12 |
| *Pectobacterium quasiaquaticum* | 4 | 0.04 | 8 | 0.19 | 6 | 0.36 |
| *Afipia felis* | 4 | 0.04 | 2 | 0.05 | 1 | 0.06 |
| *Acidimicrobium* sp. | 7 | 0.07 | 2 | 0.05 | 3 | 0.18 |
| *Schlegelella* sp. | 4 | 0.04 | 8 | 0.19 | 8 | 0.49 |
| *Geobacter* sp. | 6 | 0.06 | 5 | 0.12 | 1 | 0.06 |
| *Byssovorax cruenta* | 3 | 0.03 | 12 | 0.28 | 6 | 0.36 |
| *Cohnella* sp. | 14 | 0.14 | - | - | 1 | 0.06 |
| *Massilia timonae* | 15 | 0.14 | 2 | 0.05 | - | - |
| *Blastococcus aggregatus* | 9 | 0.09 | 2 | 0.05 | - | - |
| *Atopobium rimae* | 19 | 0.18 | - | - | 2 | 0.12 |
| *Bradyrhizobium* sp. | 8 | 0.08 | 2 | 0.05 | - | - |
| *Sporobacter termitidis* | 6 | 0.06 | 6 | 0.14 | 2 | 0.12 |
| *Terrimonas ferruginea* | 3 | 0.03 | 2 | 0.05 | 6 | 0.36 |
| *Clostridium thermosuccinogenes* | 4 | 0.04 | 2 | 0.05 | 1 | 0.06 |
| *Bryocella elongata* | 8 | 0.08 | 4 | 0.10 | - | - |
| *Thermovum composti* | 3 | 0.03 | 4 | 0.09 | 5 | 0.30 |
| *Robinsoniella peoriensis* | 3 | 0.03 | 4 | 0.09 | 6 | 0.36 |
| *Aurantimonas altamirensis* | 3 | 0.03 | 2 | 0.05 | 1 | 0.06 |
| *Actinoallomurus* sp. | 2 | 0.02 | 2 | 0.05 | - | - |
| *Chelatococcus* sp. | 2 | 0.02 | 2 | 0.05 | 6 | 0.36 |
| *Mesorhizobium huakuii* | 5 | 0.05 | 3 | 0.07 | 1 | 0.06 |
| *Synergistes* sp. | 1 | 0.01 | 2 | 0.05 | - | - |
| *Cupriavidus* sp. | 1 | 0.01 | 2 | 0.05 | 2 | 0.12 |
| *Methylibium* | 4 | 0.04 | 4 | 0.09 | 2 | 0.12 |
| *Rhizobium multihospitium* | 1 | 0.01 | 2 | 0.05 | 1 | 0.06 |
| *Desulfuromonas michiganensis* | 1 | 0.01 | 1 | 0.02 | - | - |
| *Methyloferula stellata* | 1 | 0.01 | 2 | 0.05 | - | - |
| *Kitasatospora* sp. | 1 | 0.01 | 2 | 0.05 | - | - |
| *Curtobacterium* sp. | 1 | 0.01 | 4 | 0.09 | 5 | 0.30 |
| *Mycobacterium llatzerense* | 1 | 0.01 | - | - | - | - |
| *Desulfoglaeba alkanexedens* | 1 | 0.01 | 2 | 0.05 | - | - |
| *Ruminococcus albus* | 1 | 0.01 | 2 | 0.05 | 1 | 0.06 |
| *Acidopila rosea* | 1 | 0.01 | 2 | 0.05 | 4 | 0.24 |
| *Phytohabitans suffuscus* | 1 | 0.01 | 2 | 0.05 | - | - |
| *Nitrosomonas cryotolerans* | 1 | 0.01 | 2 | 0.05 | - | - |
| *Bacillus megaterium* | 21 | 0.20 | 4 | 0.09 | 2 | 0.12 |
| *Streptomyces* sp. | 10 | 0.10 | 8 | 0.19 | 5 | 0.30 |
| *Massilia haematophila* | 7 | 0.07 | 2 | 0.05 | 1 | 0.06 |
| *Bacillus asahii* | 7 | 0.07 | 4 | 0.09 | 2 | 0.12 |
| *Acidicapsa borealis* | 4 | 0.04 | 2 | 0.05 | 1 | 0.06 |
| *Clostridium aminophilum* | 4 | 0.04 | 2 | 0.05 | - | - |
| *Methylobacterium adhaesivum* | 3 | 0.03 | 2 | 0.05 | 1 | 0.06 |
| *Clostridium subterminale* | 3 | 0.03 | 2 | 0.05 | 2 | 0.12 |
| *Arthrobacter nitroguajacolicus* | 3 | 0.03 | 2 | 0.05 | - | - |
| *Clostridium hathewayi* | 2 | 0.02 | 2 | 0.05 | 1 | 0.06 |
| *Alistipes massiliensis* | 2 | 0.02 | 2 | 0.05 | - | - |
| *Micromonospora chalcea* | 2 | 0.02 | 1 | 0.02 | - | - |
| *Belnapia moabensis* | 2 | 0.02 | 1 | 0.02 | 1 | 0.06 |
| *Oceanibaculum indicum* | 2 | 0.02 | 2 | 0.05 | - | - |
| *Mycobacterium arupense* | 2 | 0.02 | 2 | 0.05 | 1 | 0.06 |
| *Clostridium colinum* | 1 | 0.01 | 2 | 0.05 | - | - |
| *Sphingomonas mali* | 1 | 0.01 | 2 | 0.05 | - | - |
| *Nocardioides bigeumensis* | 1 | 0.01 | 4 | 0.09 | 1 | 0.06 |
| *Burkholderia tuberum* | 1 | 0.01 | 4 | 0.09 | 2 | 0.12 |
| *Aquicella* sp. | 1 | 0.01 | 2 | 0.05 | - | - |
| *Chitinimonas koreensis* | 2 | 0.02 | 4 | 0.09 | 2 | 0.12 |
| *Xenophilus aerolatus* | 2 | 0.02 | 2 | 0.05 | 1 | 0.06 |
| *Hyphomicrobium zavarzinii* | 1 | 0.01 | 2 | 0.05 | 1 | 0.06 |
| *Singulisphaera* sp. | 1 | 0.01 | 2 | 0.05 | 2 | 0.12 |
| *Thermoanaerobacterium thermosaccharolyticum* | 4 | 0.04 | 2 | 0.05 | - | - |
| *Phycicoccus* sp. | 6 | 0.06 | 2 | 0.05 | - | - |
| *Selenomonas ruminantium* | 3 | 0.03 | 2 | 0.05 | - | - |
| *Balneimonas* sp. | 2 | 0.02 | 2 | 0.05 | - | - |
| *Renibacterium* sp. | 3 | 0.03 | 2 | 0.05 | - | - |
| *Syntrophococcus sucromutans* | 1 | 0.01 | 1 | 0.02 | - | - |
| *Fibrobacter succinogenes* | 3 | 0.03 | 1 | 0.02 | - | - |
| *Ruminococcus albus* | 4 | 0.04 | 1 | 0.02 | - | - |
| *Succiniclasticum* sp. | 2 | 0.02 | 1 | 0.02 | - | - |
| *Lysinibacillus massiliensis* | 3 | 0.03 | 1 | 0.02 | - | - |
| *Pullulanibacillus* sp. | 3 | 0.03 | 1 | 0.02 | - | - |
| *Prevotella copri* | 2 | 0.02 | 1 | 0.02 | - | - |
| *Ammoniphilus* sp. | 1 | 0.01 | - | - | - | - |
| *Victivallis vadensis* | 1 | 0.01 | - | - | - | - |
| *Papillibacter cinnamivorans* | 1 | 0.01 | - | - | 6 | 0.36 |
| *Bacillus foraminis* | 1 | 0.01 | - | - | - | - |
| *Paucimonas lemoignei* | 1 | 0.01 | - | - | - | - |
| *Barnesiella viscericola* | 1 | 0.01 | - | - | - | - |
|  | **10,357** | **100.00** | **4,223** | **100.00** | **1,644** | **100.00** |
