# Supplementary material for: Effects of pesticide application on soil bacteria community structure in a cabbage-based agroecosystem in Ghana
Source: PLoS One. 2025 May 29;20(5):e0323936. doi: 10.1371/journal.pone.0323936 (PMC12121791; doi:10.1371/journal.pone.0323936)
Supplement: S2 Table — (DOCX) [file pone.0323936.s002.docx]

**SUPPLEMENTRY DATA**

**S2 Table: Taxonomic hierarchy of bacteria genus within the non-contaminated (NCS), abandoned pesticide-contaminated (AB-PCS) and active pesticide-contaminated (AC-PCS) soils.**

|  | **Treatment** | | | | | |
| --- | --- | --- | --- | --- | --- | --- |
| **Genus** | **NCS** | **Percentage** | **AB-PCS** | **Percentage** | **AC-PCS** | **Percentage** |
| Unknown | 5844 | 64.22 | 3599 | 81.61 | 3202 | 81.54 |
| *Bacillus* | 899 | 9.88 | 42 | 0.95 | 20 | 0.51 |
| *Pseudomonas* | 569 | 6.25 | 42 | 0.95 | 35 | 0.89 |
| *Prevotella* | 485 | 5.33 | 15 | 0.34 | 7 | 0.18 |
| *Rhodoplanes* | 154 | 1.69 | 108 | 2.45 | 178 | 4.53 |
| *Gemmata* | 143 | 1.57 | 103 | 2.34 | 100 | 2.55 |
| *Nostocoida* | 125 | 1.37 | 93 | 2.11 | 69 | 1.76 |
| *Nitrospira* | 37 | 0.41 | 37 | 0.84 | 47 | 1.20 |
| *Arthrobacter* | 149 | 1.64 | 42 | 0.95 | 2 | 0.05 |
| *Paenibacillus* | 91 | 1.00 | 21 | 0.48 | 11 | 0.28 |
| *Pedomicrobium* | 8 | 0.09 | 29 | 0.66 | 22 | 0.56 |
| *Selenomonas* | 70 | 0.77 | 2 | 0.05 | - | - |
| *Clostridium* | 49 | 0.54 | 21 | 0.48 | 7 | 0.18 |
| *Streptomyces* | 26 | 0.29 | 10 | 0.23 | 5 | 0.13 |
| *Singulisphaera* | 26 | 0.29 | 21 | 0.48 | 31 | 0.79 |
| *Planctomyces* | 13 | 0.14 | 21 | 0.48 | 16 | 0.41 |
| *Cystobacter* | 4 | 0.04 | 19 | 0.43 | 4 | 0.10 |
| *Massilia* | 34 | 0.37 | 8 | 0.18 | 4 | 0.10 |
| *Atopobium* | 24 | 0.26 | 2 | 0.05 | 9 | 0.23 |
| *Schlegelella* | 3 | 0.03 | 3 | 0.07 | 3 | 0.08 |
| *Afipia* | 8 | 0.09 | 5 | 0.11 | 4 | 0.10 |
| *Candidatus* | 15 | 0.16 | 10 | 0.23 | 7 | 0.18 |
| *Eggerthella* | 18 | 0.20 | 2 | 0.05 | - | - |
| *Hyphomicrobium* | 12 | 0.13 | 2 | 0.05 | 4 | 0.10 |
| *Alicyclobacillus* | 26 | 0.29 | 4 | 0.09 | 2 | 0.05 |
| *Treponema* | 19 | 0.21 | 3 | 0.07 | - | - |
| *Ruminococcus* | 15 | 0.16 | 2 | 0.05 | - | - |
| *Byssovorax* | 5 | 0.05 | 10 | 0.23 | 2 | 0.05 |
| *Solirubrobacter* | 12 | 0.13 | 4 | 0.09 | 11 | 0.28 |
| *Cohnella* | 12 | 0.13 | 2 | 0.05 | - | - |
| *Nocardioides* | 15 | 0.16 | 5 | 0.11 | 11 | 0.28 |
| *Fibrobacter* | 9 | 0.10 | 2 | 0.05 | - | - |
| *Methylobacterium* | 7 | 0.08 | 2 | 0.05 | - | - |
| *Methyloferula* | 11 | 0.12 | 1 | 0.02 | 7 | 0.18 |
| *Bradyrhizobium* | 7 | 0.08 | 2 | 0.05 | 16 | 0.41 |
| *Chelatococcus* | 22 | 0.24 | 29 | 0.65 | 21 | 0.54 |
| *Pullulanibacillus* | 6 | 0.07 | 2 | 0.05 | - | - |
| *Burkholderia* | 5 | 0.05 | 3 | 0.07 | 3 | 0.08 |
| *Sporobacter* | 5 | 0.05 | 2 | 0.05 | - | - |
| *Geobacter* | 8 | 0.09 | 5 | 0.11 | 1 | 0.03 |
| *Mesorhizobium* | 8 | 0.09 | 4 | 0.09 | 5 | 0.13 |
| *Desulfuromonas* | 2 | 0.02 | 8 | 0.18 | 2 | 0.05 |
| *Acidobacterium* | 4 | 0.04 | 3 | 0.07 | 4 | 0.10 |
| *Pirellula* | 12 | 0.13 | 10 | 0.23 | 4 | 0.10 |
| *Acidicapsa* | 6 | 0.07 | 4 | 0.09 | 3 | 0.08 |
| *Methylibium* | 4 | 0.04 | 2 | 0.05 | 4 | 0.10 |
| *Thermovum* | 2 | 0.02 | 2 | 0.05 | 1 | 0.03 |
| *Terrimonas* | 3 | 0.03 | 3 | 0.07 | 2 | 0.05 |
| *Bryocella* | 3 | 0.03 | - | - | 4 | 0.10 |
| *Pseudoxanthomonas* | 3 | 0.03 | - | - | 2 | 0.05 |
| *Actinoallomurus* | 2 | 0.02 | - | - | 4 | 0.10 |
| *Paucimonas* | 4 | 0.04 | - | - | 2 | 0.05 |
| *Lysobacter* | 4 | 0.04 | - | - | 2 | 0.05 |
| *Mycobacterium* | 7 | 0.08 | - | - | 2 | 0.05 |
| *Balneimonas* | 13 | 0.14 | - | - | 2 | 0.05 |
| *Alistipes* | 2 | 0.02 | - | - | 2 | 0.05 |
| *Kitasatospora* | 1 | 0.01 | - | - | 2 | 0.05 |
| *Bacteroides* | 7 | 0.08 | - | - | 4 | 0.10 |
| *Synergistes* | 1 | 0.01 | - | - | 2 | 0.05 |
| *Cupriavidus* | 1 | 0.01 | - | - | 7 | 0.18 |
| *Rhizobium* | 2 | 0.02 | - | - | 2 | 0.05 |
| *Micromonospora* | 2 | 0.02 | 2 | 0.05 | - | - |
| *Aurantimonas* | 2 | 0.02 | 2 | 0.05 | - | - |
| *Belnapia* | 2 | 0.02 | 2 | 0.05 | - | - |
| *Rhodococcus* | 2 | 0.02 | 3 | 0.07 | - | - |
| *Georgfuchsia* | 1 | 0.01 | 5 | 0.11 | - | - |
| *Curtobacterium* | 1 | 0.01 | 3 | 0.07 | - | - |
| *Nostoc* | 1 | 0.01 | 3 | 0.07 | - | - |
| *Novosphingobium* | 1 | 0.01 | 2 | 0.05 | - | - |
| *Acidimicrobium* | 1 | 0.01 | 3 | 0.07 | - | - |
| *Acidovorax* | 1 | 0.01 | 3 | 0.07 | - | - |
| *Aquicella* | 1 | 0.01 | 2 | 0.05 | - | - |
| *Acidopila* | 1 | 0.01 | 2 | 0.05 | - | - |
| *Intrasporangium* | 1 | 0.01 | 2 | 0.05 | - | - |
| *Phytohabitans* | 1 | 0.01 | - | - | 4 | 0.10 |
| *Nitrosomonas* | 1 | 0.01 | 5 | 0.11 | - | - |
|  | **9,100** | **100.00** | **4,410** | **100.00** | **3,927** | **100.00** |
