# Supplementary material for: Effects of pesticide application on soil bacteria community structure in a cabbage-based agroecosystem in Ghana
Source: PLoS One. 2025 May 29;20(5):e0323936. doi: 10.1371/journal.pone.0323936 (PMC12121791; doi:10.1371/journal.pone.0323936)
Supplement: S3 Table — (DOCX) [file pone.0323936.s003.docx]

**SUPPLEMENTARY DATA**

**S3 Table: Taxonomic hierarchy of bacteria family within the non-contaminated (NCS), abandoned pesticide-contaminated (AB-PCS) and active pesticide-contaminated (AC-PCS) soils.**

|  | **Treatment** | | | | | |
| --- | --- | --- | --- | --- | --- | --- |
| **Family** | **NCS** | **Percentage** | **AB-PCS** | **Percentage** | **AC-PCS** | **Percentage** |
| Unknown | 4305 | 26.78 | 3910 | 59.12 | 2598 | 55.93 |
| *Gemmataceae* | 1374 | 8.55 | 978 | 14.79 | 701 | 15.09 |
| *Veillonellaceae* | 1775 | 11.04 | 4 | 0.06 | 3 | 0.06 |
| *Micrococcaceae* | 1747 | 10.87 | 6 | 0.09 | 2 | 0.04 |
| *Bacillaceae* | 1554 | 9.67 | 34 | 0.51 | 14 | 0.30 |
| *Pseudomonadaceae* | 925 | 5.75 | 7 | 0.11 | 3 | 0.06 |
| *Prevotellaceae* | 872 | 5.42 | 9 | 0.14 | 6 | 0.13 |
| *Lachnospiraceae* | 509 | 3.17 | 8 | 0.12 | 5 | 0.11 |
| *Oxalobacteraceae* | 481 | 2.99 | 9 | 0.14 | 11 | 0.24 |
| *Ruminococcaceae* | 245 | 1.52 | 8 | 0.12 | 1 | 0.02 |
| *Isosphaeraceae* | 202 | 1.26 | 109 | 1.65 | 76 | 1.64 |
| *Paenibacillaceae* | 195 | 1.21 | 15 | 0.23 | 6 | 0.13 |
| *Gaiellaceae* | 132 | 0.82 | 19 | 0.29 | 29 | 0.62 |
| *Coriobacteriaceae* | 122 | 0.76 | 7 | 0.11 | 9 | 0.19 |
| *Hyphomicrobiaceae* | 121 | 0.75 | 233 | 3.52 | 245 | 5.27 |
| *Acidobacteriaceae* | 77 | 0.48 | 29 | 0.44 | 45 | 0.97 |
| *Pirellulaceae* | 41 | 0.26 | 136 | 2.06 | 90 | 1.94 |
| *Comamonadaceae* | 51 | 0.32 | 126 | 1.91 | 67 | 1.44 |
| *Syntrophobacteraceae* | 33 | 0.21 | 96 | 1.45 | 55 | 1.18 |
| *Nitrospiraceae* | 23 | 0.14 | 25 | 0.38 | 43 | 0.93 |
| *Bradyrhizobiaceae* | 68 | 0.42 | 29 | 0.44 | 32 | 0.69 |
| *Rhodospirillaceae* | 66 | 0.41 | 29 | 0.44 | 28 | 0.60 |
| *Beijerinckiaceae* | 27 | 0.17 | 15 | 0.23 | 13 | 0.28 |
| *Phyllobacteriaceae* | 18 | 0.11 | 12 | 0.18 | 18 | 0.39 |
| *Cystobacteraceae* | 14 | 0.09 | 47 | 0.71 | 39 | 0.84 |
| *Polyangiaceae* | 19 | 0.12 | 18 | 0.27 | 6 | 0.13 |
| *Streptomycetaceae* | 55 | 0.34 | 14 | 0.21 | 28 | 0.60 |
| *Intrasporangiaceae* | 72 | 0.45 | 5 | 0.08 | 2 | 0.04 |
| *Planctomycetaceae* | 12 | 0.07 | 15 | 0.23 | 8 | 0.17 |
| *Solirubrobacteraceae* | 24 | 0.15 | 5 | 0.08 | 11 | 0.24 |
| *Xanthomonadaceae* | 38 | 0.24 | 23 | 0.35 | 16 | 0.34 |
| *Neisseriaceae* | 8 | 0.05 | 23 | 0.35 | 3 | 0.06 |
| *Koribacteraceae* | 52 | 0.32 | 16 | 0.24 | 6 | 0.13 |
| *Micromonosporaceae* | 50 | 0.31 | 12 | 0.18 | 14 | 0.30 |
| *Clostridiaceae* | 40 | 0.25 | 8 | 0.12 | 3 | 0.06 |
| *Nitrosomonadaceae* | 3 | 0.02 | 5 | 0.08 | 1 | 0.02 |
| *Sinobacteraceae* | 4 | 0.02 | 6 | 0.09 | 3 | 0.06 |
| *Thermomonosporaceae* | 7 | 0.04 | 2 | 0.03 | 6 | 0.13 |
| *Rhizobiaceae* | 23 | 0.14 | 5 | 0.08 | 8 | 0.17 |
| *Nocardioidaceae* | 23 | 0.14 | 7 | 0.11 | 9 | 0.19 |
| *Acetobacteraceae* | 30 | 0.19 | 16 | 0.24 | 11 | 0.24 |
| *Chitinophagaceae* | 20 | 0.12 | 7 | 0.11 | 4 | 0.09 |
| *Xanthobacteraceae* | 15 | 0.09 | 7 | 0.11 | 3 | 0.06 |
| *Pelobacteraceae* | 3 | 0.02 | 5 | 0.08 | 1 | 0.02 |
| *Haliangiaceae* | 2 | 0.01 | 1 | 0.02 | 2 | 0.04 |
| *Alicyclobacillaceae* | 42 | 0.26 | 1 | 0.02 | 2 | 0.04 |
| *Spirochaetaceae* | 35 | 0.22 | 7 | 0.11 | 5 | 0.11 |
| *Coxiellaceae* | 3 | 0.02 | 2 | 0.03 | 1 | 0.02 |
| *Fibrobacteraceae* | 15 | 0.09 | 2 | 0.03 | 7 | 0.15 |
| *Sphingomonadaceae* | 17 | 0.11 | 9 | 0.14 | 10 | 0.22 |
| *Methylobacteriaceae* | 12 | 0.07 | 14 | 0.21 | 18 | 0.39 |
| *Geodermatophilaceae* | 27 | 0.17 | 2 | 0.03 | 14 | 0.30 |
| *Nostocaceae* | 5 | 0.03 | 7 | 0.11 | 3 | 0.06 |
| *Aurantimonadaceae* | 10 | 0.06 | 7 | 0.11 | 4 | 0.08 |
| *Desulfobulbaceae* | 6 | 0.04 | 2 | 0.03 | 39 | 0.84 |
| *Mycobacteriaceae* | 12 | 0.07 | 9 | 0.14 | 5 | 0.11 |
| *Nocardiaceae* | 7 | 0.04 | 5 | 0.08 | 2 | 0.04 |
| *Conexibacteraceae* | 9 | 0.06 | 2 | 0.03 | 1 | 0.02 |
| *Peptostreptococcaceae* | 10 | 0.06 | 5 | 0.08 | 6 | 0.13 |
| *Sporolactobacillaceae* | 10 | 0.06 | 2 | 0.03 | 5 | 0.11 |
| *Anaerolinaceae* | 4 | 0.02 | 7 | 0.11 | 9 | 0.19 |
| *Microbulbiferaceae* | 3 | 0.02 | 9 | 0.14 | 2 | 0.04 |
| *Bacteriophage* | 3 | 0.02 | 7 | 0.11 | 12 | 0.26 |
| *Acidimicrobiaceae* | 2 | 0.01 | 5 | 0.08 | 13 | 0.28 |
| *Rhodobacteraceae* | 2 | 0.01 | 2 | 0.03 | 1 | 0.02 |
| *Streptosporangiaceae* | 11 | 0.07 | 9 | 0.14 | 7 | 0.15 |
| *Caulobacteraceae* | 10 | 0.06 | 3 | 0.05 | 2 | 0.04 |
| *Providencia thailandensis* | 2 | 0.01 | 5 | 0.08 | 3 | 0.06 |
| *Solibacteraceae* | 3 | 0.02 | 7 | 0.11 | 7 | 0.15 |
| *Rikenellaceae* | 3 | 0.02 | 5 | 0.08 | 2 | 0.04 |
| *Synergistaceae* | 3 | 0.02 | 7 | 0.11 | 9 | 0.19 |
| *Bacteroidaceae* | 16 | 0.10 | 12 | 0.18 | 5 | 0.11 |
| *Frankiaceae* | 16 | 0.10 | 37 | 0.56 | 7 | 0.15 |
| *Microbacteriaceae* | 15 | 0.09 | 35 | 0.53 | 7 | 0.15 |
| *Burkholderiaceae* | 0 | 0.00 | 5 | 0.08 | 13 | 0.28 |
| *Thermoactinomycetaceae* | 0 | 0.00 | 2 | 0.03 | 7 | 0.15 |
| *Geobacteraceae* | 0 | 0.00 | 12 | 0.18 | 7 | 0.15 |
| *Nocardiopsaceae* | 0 | 0.00 | 2 | 0.03 | 7 | 0.15 |
| *Dethiosulfovibrionaceae* | 34 | 0.21 | 29 | 0.44 | 17 | 0.37 |
| *Planococcaceae* | 75 | 0.47 | 65 | 0.98 | 15 | 0.32 |
| *Actinosynnemataceae* | 17 | 0.11 | 20 | 0.30 | 9 | 0.19 |
| *Myxococcaceae* | 14 | 0.09 | 23 | 0.35 | 13 | 0.28 |
| *Victivallaceae* | 8 | 0.05 | 9 | 0.14 | 7 | 0.15 |
| *Burkholderiaceae* | 8 | 0.05 | 6 | 0.09 | 7 | 0.15 |
| *Geobacteraceae* | 8 | 0.05 | 5 | 0.08 | 3 | 0.06 |
| *Erysipelotrichaceae* | 7 | 0.04 | 4 | 0.06 | 3 | 0.06 |
| *Christensenellaceae* | 6 | 0.04 | 4 | 0.06 | 3 | 0.06 |
| *Aeromicrobium yanjiei* | 6 | 0.04 | 4 | 0.06 | 3 | 0.06 |
| *Pseudonocardiaceae* | 5 | 0.03 | 3 | 0.05 | 3 | 0.06 |
| *Promicromonosporaceae* | 10 | 0.06 | 4 | 0.06 | 6 | 0.12 |
| *Phormidiaceae* | 4 | 0.02 | 4 | 0.06 | 3 | 0.06 |
| *Rhodocyclaceae* | 4 | 0.02 | 4 | 0.06 | 3 | 0.06 |
| *Kineosporiaceae* | 4 | 0.02 | 2 | 0.03 | 2 | 0.04 |
| *Salmonella enterica* | 4 | 0.02 | 2 | 0.03 | 2 | 0.04 |
| *Patulibacteraceae* | 4 | 0.02 | 3 | 0.05 | 2 | 0.04 |
| *Propionibacteriaceae* | 3 | 0.02 | 2 | 0.03 | 2 | 0.04 |
| *Sphingobacteriaceae* | 3 | 0.02 | 7 | 0.11 | 2 | 0.04 |
| *Symbiobacteriaceae* | 2 | 0.01 | 3 | 0.05 | 2 | 0.04 |
| *Marinilabiaceae* | 2 | 0.01 | 1 | 0.02 | 2 | 0.04 |
| *Desulfuromonadaceae* | 2 | 0.01 | 1 | 0.02 | 2 | 0.04 |
| *Acinetobacter lactucae* | 2 | 0.01 | 3 | 0.05 | 2 | 0.04 |
| *Methylocystaceae* | 2 | 0.01 | 3 | 0.05 | 2 | 0.04 |
| *Alcaligenaceae* | 2 | 0.01 | 3 | 0.05 | 2 | 0.04 |
| *Pseudanabaenaceae* | 4 | 0.02 | 5 | 0.08 | 2 | 0.04 |
| *Rhodocyclaceae* | - | - | 6 | 0.09 | - | - |
| *Micromonosporaceae* | 2 | 0.01 | 4 | 0.06 | - | - |
| *Ectothiorhodospiraceae* | 2 | 0.01 | 2 | 0.03 | - | - |
| *Methylomirabiliaceae* | 2 | 0.01 | 3 | 0.05 | - | - |
| *Salmonella enterica* | 3 | 0.02 | 4 | 0.06 | - | - |
| *Hydrogenophilaceae* | 3 | 0.02 | 5 | 0.08 | - | - |
| *Solibacteraceae* | - | - | 2 | 0.03 | - | - |
| *Methylobacteriaceae* | 1 | 0.01 | 3 | 0.05 | - | - |
| *Cytophagaceae* | 1 | 0.01 | 5 | 0.08 | - | - |
| *Syntrophaceae* | - | - | 1 | 0.02 | - | - |
| *Erythrobacteraceae* | 1 | 0.01 | 2 | 0.03 | - | - |
| *Methylococcaceae* | 3 | 0.02 | 2 | 0.03 | - | - |
| *Alcaligenaceae* | 2 | 0.01 | 2 | 0.03 | - | - |
| *Desulfobacteraceae* | 2 | 0.01 | 2 | 0.03 | - | - |
| *Desulfovibrionaceae* | 2 | 0.01 | 2 | 0.03 | - | - |
| *Holophagaceae* | 2 | 0.01 | 1 | 0.02 | - | - |
| *Frankiaceae* | 2 | 0.01 | 1 | 0.02 | - | - |
| *Acidimicrobiaceae* | 1 | 0.01 | 1 | 0.02 | - | - |
| *Rubrobacteraceae* | 1 | 0.01 | - | - | - | - |
| *Bacteriophage* | 1 | 0.01 | - | - | - | - |
| *Anaerolinaceae* | 1 | 0.01 | - | - | - | - |
| *Piscirickettsiaceae* | 1 | 0.01 | - | - | - | - |
| *Nectriaceae* | 1 | 0.01 | - | - | - | - |
| *Chthonomonadaceae* | 1 | 0.01 | - | - | - | - |
| *Gemmatimonadaceae* | 1 | 0.01 | - | - | - | - |
| *Rhodobiaceae* | 1 | 0.01 | - | - | - | - |
| *Peptococcaceae* | 1 | 0.01 | - | - | - | - |
| *Caldilineaceae* | 1 | 0.01 | - | - | - | - |
| *Brocadiaceae* | 1 | 0.01 | - | - | - | - |
| *Rhodobacteraceae* | 1 | 0.01 | - | - | - | - |
| *Ardenscatenaceae* | 1 | 0.01 | - | - | - | - |
| *Flavobacteriaceae* | 1 | 0.01 | - | - | - | - |
| *Mycobacteriaceae* | 1 | 0.01 | - | - | - | - |
|  | **16,074** | **100.00** | **6,614** | **100.00** | **4,645** | **100.00** |
