# Supplementary material for: Effects of pesticide application on soil bacteria community structure in a cabbage-based agroecosystem in Ghana
Source: PLoS One. 2025 May 29;20(5):e0323936. doi: 10.1371/journal.pone.0323936 (PMC12121791; doi:10.1371/journal.pone.0323936)
Supplement: S4 Table — (DOCX) [file pone.0323936.s004.docx]

**SUPPLEMENTARY DATA**

**S4 Table: Taxonomic hierarchy of bacteria order within the non-contaminated (NCS), abandoned pesticide-contaminated (AB-PCS) and active pesticide-contaminated (AC-PCS) soils.**

|  | **Treatment** | | | | | |
| --- | --- | --- | --- | --- | --- | --- |
| **Order** | **NCS** | **Percentage** | **AB-PCS** | **Percentage** | **AC-PCS** | **Percentage** |
| Unknown | 1187 | 7.89 | 680 | 29.25 | 240 | 12.62 |
| *Clostridiales* | 3388 | 22.53 | 39 | 1.68 | 17 | 0.89 |
| *Bacteroidales* | 2396 | 15.93 | 20 | 0.86 | 19 | 1.00 |
| *Actinomycetales* | 2261 | 15.03 | 90 | 3.87 | 99 | 5.21 |
| *Bacillales* | 1983 | 13.19 | 62 | 2.67 | 29 | 1.52 |
| *Pseudomonadales* | 931 | 6.19 | 35 | 1.50 | 14 | 0.74 |
| *Gemmatales* | 597 | 3.97 | 426 | 18.32 | 368 | 19.35 |
| *Burkholderiales* | 558 | 3.70 | 82 | 3.52 | 38 | 2.00 |
| *Rhizobiales* | 467 | 3.11 | 331 | 14.24 | 573 | 30.13 |
| *Acidobacteriales* | 133 | 0.88 | 36 | 1.55 | 35 | 1.84 |
| *Rhodospirillales* | 111 | 0.74 | 48 | 2.06 | 42 | 2.21 |
| *Nitrospirales* | 61 | 0.41 | 86 | 3.70 | 163 | 8.57 |
| *Myxococcales* | 91 | 0.61 | 88 | 3.78 | 66 | 3.47 |
| *Pirellulales* | 41 | 0.27 | 58 | 2.49 | 34 | 1.79 |
| *Gaiellales* | 139 | 0.92 | 23 | 0.99 | 34 | 1.79 |
| *Syntrophobacterales* | 33 | 0.22 | 43 | 1.85 | 24 | 1.26 |
| *Solirubrobacterales* | 80 | 0.53 | 8 | 0.34 | 18 | 0.95 |
| *Xanthomonadales* | 46 | 0.31 | 17 | 0.73 | 9 | 0.47 |
| *Coriobacteriales* | 122 | 0.81 | 3 | 0.13 | 5 | 0.26 |
| *Enterobacterales* | 46 | 0.31 | 1 | 0.04 | - | - |
| *Spirochaetales* | 35 | 0.23 | 3 | 0.13 | - | - |
| *Acidimicrobiales* | 24 | 0.16 | 10 | 0.43 | 15 | 0.79 |
| *Desulfuromonadales* | 23 | 0.15 | 16 | 0.69 | 5 | 0.26 |
| *Sphingomonadales* | 18 | 0.12 | 5 | 0.22 | - | - |
| *Fibrobacterales* | 15 | 0.10 | 1 | 0.04 | - | - |
| *Gemmatimonadales* | 15 | 0.10 | 4 | 0.17 | 1 | 0.05 |
| *Solibacterales* | 15 | 0.10 | 7 | 0.30 | 4 | 0.21 |
| *Planctomycetales* | 12 | 0.08 | 14 | 0.60 | 8 | 0.42 |
| *Caulobacterales* | 10 | 0.07 | - | - | 4 | 0.21 |
| *Neisseriales* | 11 | 0.07 | 15 | 0.65 | 6 | 0.32 |
| *Chromatiales* | - | - | 7 | 0.30 | 1 | 0.05 |
| *Pasteurellales* | 15 | 0.10 | 2 | 0.09 | 4 | 0.21 |
| *Halobacteriales* | 7 | 0.05 | 10 | 0.43 | 2 | 0.11 |
| *Thermogemmatisporales* | 3 | 0.02 | 3 | 0.13 | 3 | 0.16 |
| *Nitrosomonadales* | 3 | 0.02 | 5 | 0.22 | 1 | 0.05 |
| *Rhodobacterales* | 3 | 0.02 | 6 | 0.26 | 2 | 0.11 |
| *Phycisphaerales* | 2 | 0.01 | 5 | 0.22 | 2 | 0.11 |
| *Anaerolineales* | 4 | 0.03 | 3 | 0.13 | 2 | 0.11 |
| *Nostocales* | 5 | 0.03 | 3 | 0.13 | - | - |
| *Desulfobacterales* | 4 | 0.03 | 2 | 0.09 | - | - |
| *Legionellales* | 4 | 0.03 | 3 | 0.13 | 1 | 0.05 |
| *Sphingobacteriales* | 3 | 0.02 | 3 | 0.13 | - | - |
| *Hyphomicrobiales* | - | - | 2 | 0.09 | 2 | 0.11 |
| *Cytophagales* | 3 | 0.02 | 3 | 0.13 | - | - |
| *Rickettsiales* | 28 | 0.19 | - | - | - | - |
| *Victivallales* | 31 | 0.21 | - | - | - | - |
| *Erysipelotrichales* | 8 | 0.05 | - | - | - | - |
| *Oscillatoriales* | 4 | 0.03 | - | - | - | - |
| *Acholeplasmatales* | 4 | 0.03 | 2 | 0.09 | - | - |
| *Pelagibacterales* | 3 | 0.02 | 1 | 0.04 | 9 | 0.47 |
| *Methylomirabiliales* | 3 | 0.02 | - | - | - | - |
| *Hydrogenophilales* | - | - | 4 | 0.18 | - | - |
| *Methylococcales* | - | - | 1 | 0.04 | - | - |
| *Desulfovibrionales* | - | - | 2 | 0.08 | - | - |
| *Holophagales* | - | - | - | - | - | - |
| *Chloroflexales* | - | - | 1 | 0.04 | - | - |
| *Nanopelagicales* | 1 | 0.01 | - | - | - | - |
| *Lactobacillales* | 1 | 0.01 | - | - | - | - |
| *Micrococcales* | - | - |  | - | 2 | 0.11 |
| *Thiotrichales* | 1 | 0.01 | - | - | - | - |
| *Chthonomonadales* | 1 | 0.01 | - | - | - | - |
| *Caldilineales* | 1 | 0.01 | - | - | - | - |
| *Brocadiales* | 1 | 0.01 | - | - | - | - |
| *Ardenscatenales* | 1 | 0.01 | - | - | - | - |
| *Flavobacteriales* | 1 | 0.01 | - | - | - | - |
|  | **15,039** | **100.00** | **2,325** | **100.00** | **1,902** | **100.00** |
