# Supplementary material for: Effects of pesticide application on soil bacteria community structure in a cabbage-based agroecosystem in Ghana
Source: PLoS One. 2025 May 29;20(5):e0323936. doi: 10.1371/journal.pone.0323936 (PMC12121791; doi:10.1371/journal.pone.0323936)
Supplement: S5 Table — (DOCX) [file pone.0323936.s005.docx]

**SUPPLEMENTARY DATA**

**S5 Table: Taxonomic hierarchy of bacteria class within the non-contaminated (NCS), abandoned pesticide-contaminated (AB-PCS) and active pesticide-contaminated (AC-PCS) soils.**

|  | **Treatment** | | | | | |
| --- | --- | --- | --- | --- | --- | --- |
| **Class** | **NCS** | **Percentage** | **AB-PCS** | **Percentage** | **AC-PCS** | **Percentage** |
| Unknown | 650 | 5.02 | 520 | 16.63 | 327 | 10.98 |
| *Clostridia* | 2941 | 22.73 | 54 | 1.73 | 18 | 0.60 |
| *Bacteroidia* | 2069 | 15.99 | 26 | 0.83 | 13 | 0.44 |
| *Actinobacteria* | 1938 | 14.98 | 92 | 2.94 | 119 | 4.00 |
| *Bacilli* | 1722 | 13.30 | 278 | 8.90 | 117 | 4.27 |
| *Gammaproteobacteria* | 856 | 6.80 | 89 | 2.85 | 316 | 10.61 |
| *Alphaproteobacteria* | 602 | 4.66 | 547 | 17.50 | 877 | 29.45 |
| *Betaproteobacteria* | 585 | 4.52 | 367 | 11.74 | - | - |
| *Planctomycetia* | 574 | 4.44 | 498 | 15.93 | 321 | 10.78 |
| *Thermoleophilia* | 190 | 1.47 | 41 | 1.31 | 132 | 4.43 |
| *Deltaproteobacteria* | 173 | 1.34 | 154 | 4.93 | 80 | 2.69 |
| *Acidobacteria* | 220 | 1.70 | 203 | 6.49 | 186 | 6.25 |
| *Nitrospira* | 53 | 0.41 | 110 | 3.52 | 290 | 9.74 |
| *Coriobacteriia* | 105 | 0.81 | 4 | 0.13 | 13 | 0.44 |
| *Synergistia* | 37 | 0.29 | - | - | 3 | 0.10 |
| *Gemmatimonadetes* | 34 | 0.26 | 23 | 0.74 | 15 | 0.50 |
| *Spirochaetes* | 31 | 0.24 | 4 | 0.13 | - | - |
| *Acidimicrobiia* | 21 | 0.16 | 13 | 0.42 | 40 | 1.34 |
| *Anaerolineae* | 17 | 0.13 | 37 | 1.18 | 24 | 0.81 |
| *Ktedonobacteria* | 16 | 0.12 | 6 | 0.19 | 18 | 0.60 |
| *Fibrobacteria* | 13 | 0.10 | 3 | 0.10 | - | - |
| *Solibacteres* | 13 | 0.10 | 9 | 0.29 | 11 | 0.37 |
| *Verrucomicrobiae* | 12 | 0.09 | 1 | 0.03 | - | - |
| *Phycisphaerae* | 9 | 0.07 | 20 | 0.64 | 18 | 0.60 |
| *Chloroflexia* | 8 | 0.06 | 4 | 0.13 | 3 | 0.10 |
| *Oscillatoriophycideae* | 14 | 0.10 | - | - | - | - |
| *Nostocophycideae* | 4 | 0.03 | 4 | 0.13 | - | - |
| *Thermomicrobia* | 3 | 0.02 | 3 | 0.10 | - | - |
| *Cytophagia* | 3 | 0.02 | 4 | 0.13 | - | - |
| *Sphingobacteriia* | 3 | 0.02 | 4 | 0.13 | - | - |
| *Elusimicrobia* | 2 | 0.02 | 5 | 0.16 | - | - |
| *Mollicutes* | 6 | 0.05 | - | - | - | - |
| *Synechococcophycideae* | 4 | 0.03 | - | - | - | - |
| *Erysipelotrichi* | 6 | 0.05 | - | - | - | - |
| *Opitutae* | - | - | 1 | 0.03 | - | - |
| *Holophagae* | - | - | 1 | 0.03 | - | - |
| Sordariomycetes | - | - | 1 | 0.03 | - | - |
| *Rubrobacteria* | - | - | - | - | 8 | 0.27 |
| *Chthonomonadetes* | - | - | - | - | 3 | 0.10 |
| *Flavobacteriia* | - | - | - | - | 3 | 0.10 |
|  | **12,942** | **100.00** | **3,126** | **100.00** | **2,978** | **100.00** |
