# Supplementary material for: Effects of pesticide application on soil bacteria community structure in a cabbage-based agroecosystem in Ghana
Source: PLoS One. 2025 May 29;20(5):e0323936. doi: 10.1371/journal.pone.0323936 (PMC12121791; doi:10.1371/journal.pone.0323936)
Supplement: S6 Table — (DOCX) [file pone.0323936.s006.docx]

**S6 Table: Taxonomic hierarchy of bacteria phyla within the non-contaminated (NCS), abandoned pesticide-contaminated (AB-PCS) and active pesticide-contaminated (AC-PCS) soils.**

| **Phylum** | **Treatment** | | | | | |
| --- | --- | --- | --- | --- | --- | --- |
|  | **NCS** | **Percentage** | **AB-PCS** | **Percentage** | **AC-PCS** | **Percentage** |
| Unknown | 221 | 2.24 | 306 | 9.15 | 269 | 10.67 |
| *Firmicutes* | 3500 | 35.50 | 129 | 3.86 | 80 | 3.17 |
| *Actinobacteria* | 1746 | 17.71 | 163 | 4.87 | 224 | 8.89 |
| *Proteobacteria* | 1748 | 17.73 | 1349 | 40.34 | 936 | 37.13 |
| *Bacteroidetes* | 1634 | 16.57 | 43 | 1.29 | 31 | 1.23 |
| *Planctomycetes* | 471 | 4.78 | 717 | 21.44 | 419 | 16.62 |
| *Acidobacteria* | 241 | 2.44 | 389 | 11.63 | 293 | 11.62 |
| *Chloroflexi* | 48 | 0.49 | 68 | 2.03 | 45 | 1.79 |
| *Nitrospirae* | 49 | 0.50 | 102 | 3.05 | 126 | 5.00 |
| *Gemmatimonadetes* | 36 | 0.37 | 34 | 1.02 | 34 | 1.35 |
| *Lentisphaerae* | 38 | 0.39 | 2 | 0.06 | - | - |
| *Cyanobacteria* | 25 | 0.25 | 11 | 0.33 | 6 | 0.24 |
| *Spirochaetes* | 24 | 0.24 | 4 | 0.12 | - | - |
| *Verrucomicrobia* | 21 | 0.21 | 15 | 0.45 | 12 | 0.48 |
| *Fibrobacteres* | 10 | 0.10 | 12 | 0.36 | - | - |
| *Armatimonadetes* | 6 | 0.06 | - | - | 23 | 0.91 |
| *Elusimicrobia* | 1 | 0.01 | - | - | - | - |
| *Synergistetes* | 33 | 0.33 | - | - | 23 | 0.81 |
| *Tenericutes* | 5 | 0.05 | - | - | - | - |
| *Elusimicrobia* | 3 | 0.03 | - | - | - | - |
|  | **9,860** | **100.00** | **3,344** | **100.00** | **2,521** | **100.00** |
