# Supplementary material for: Effects of pesticide application on soil bacteria community structure in a cabbage-based agroecosystem in Ghana
Source: PLoS One. 2025 May 29;20(5):e0323936. doi: 10.1371/journal.pone.0323936 (PMC12121791; doi:10.1371/journal.pone.0323936)
Supplement: Attachment — Submitted filename: Response to reviewers comments.docx [file pone.0323936.s008.docx]

**Response to Reviewers’ Comments**

**Response to Editor and Reviewers’ Comments**

Dear Editor and Reviewers,

Thank you very much for your constructive comments. Based on the comments, we have made changes in the manuscript which are detailed below. In the revised manuscript, the corrected content and modifications to the manuscript have been highlighted in yellow.

Thank you.

| **Comments** | **Responses** |
| --- | --- |
| **Reviewer 1**   1. **Abstract:** The abstract presents an important research question but lacks conciseness and clarity. 2. To improve, the abstract should be rewritten to emphasize the major findings and their significance, while also briefly linking the changes in soil properties to microbial shifts 3. **Introduction:** The introduction provides comprehensive background information but is somewhat lengthy and contains sections that do not directly relate to the study's core focus on soil microbiomes 4. The discussion of pesticide impacts on air, water, and soil is informative, yet it would be more effective if directly tied to soil microbial health. 5. Furthermore, although the study objectives are mentioned, there is a lack of a clear hypothesis or specific research questions. 6. To improve this section, I recommend streamlining the background, focusing more on the direct relevance to soil microbiology, and defining specific hypotheses. 7. Additionally, strengthening the logical flow of language and providing clearer connections between the research objectives and methodology will improve the readability and focus of the introduction. 8. **Study Area:** The description of the study area's climate and soil characteristics is adequate, but the manuscript could benefit from more detailed information about the long-term environmental impacts of cabbage cultivation. 9. Specifically, the types of pesticides used, historical application rates, and changes in cultivation area over time are not provided. 10. **Sampling Procedure**: The sampling procedure relies on grouping sampling sites based on farmers' reported pesticide usage history. While this approach is valid, it may introduce some bias, especially if farmers' recollections or records are inaccurate. 11. Furthermore, the "non-contaminated" soil samples were exclusively collected from pristine forest reserves, which may not fully represent agricultural soils that have not been exposed to pesticides. 12. To improve generalizability, I suggest expanding the sampling strategy to include agricultural soils known to have minimal pesticide exposure, providing a more accurate comparison. 13. **Statistical Data Interpretation**: The manuscript does not elaborate on advanced ecological statistical methods such as network analysis or redundancy analysis (RDA). Including a brief explanation of additional methods, or incorporating them into the analysis, would significantly enhance the robustness of the data interpretation. 14. **Discussion:** The discussion addresses the reduced abundance of Pseudomonas and Bacillus in pesticide-contaminated soils, which contradicts previous studies. However, the manuscript does not offer a clear explanation for this discrepancy, and further exploration into possible reasons (such as pesticide type or soil conditions) is needed. 15. Additionally, some sentences are overly complex and densely packed with information, which could hinder readability. For example, the sentence, "Pseudomonas spp. are facultative anaerobes involved in several important processes..." could be simplified and split into more digestible points. 16. It is also recommended to include a section discussing the study's limitations, such as potential biases in sampling or the sequencing method, and suggesting directions for future research. | Thank you for your valuable and thoughtful comments. We have rewritten the abstract to make it more concise and clearer. This is found on page 2 highlighted in yellow  Thank you for your thoughtful comments. This comment has been addressed in comment 1. We have also made corresponding modifications to the abstract linking the changes in soil properties to microbial shifts. This is also found in page 2 highlighted in yellow  Thank you for your comments. This comment has been addressed by rewriting and removing unrelated portions to make it more focused on soil microbiomes. This is found in pages 3-5 highlighted in yellow  The discussion of pesticide impacts on air, water, and soil has been directly linked to soil microbial health as requested. This is found in page 4 highlighted in yellow  Thank you for your insightful comments. This comment has been addressed with clear hypotheses. This is found in page 5 highlighted in yellow  Thank you very much. We have streamlined the background and focused directly on soil microbiology. The hypothesis has been defined specifically.  In the revised manuscript, we have made modifications to the introduction and made it clearer between the research objectives and the methodology.  Thank you very much for this comment. This comment has been addressed and the detailed information about the long-term environmental impacts of cabbage cultivation has been addressed. This is found in page 7 and 8 highlighted in yellow  Thank you for your valuable and thoughtful comment. We have addressed this comment and provided the types of pesticides used, application rates and changes in cultivation area over time. This is found in page 7 and 8 highlighted in yellow  This comment is valuable, thank you very much. We believe this procedure is valid. But due to the possible biases in this procedure, we have included limitations on Page 29 as part of the work and proposed that future research should further investigate the reported trends or expand the specific framework used in the current research to build upon the methodology of the study.  Thank you for this valuable and thoughtful comment. The pristine forest reserve provides more details and specific information about the abundance and diversity of soil bacteria present in the study area that have not been affected by the constant application of pesticides. This information served as a baseline to the areas affected by farming activities with pesticides contamination. This comment was addressed on page 9.  Thank you very much for this comment. In this study, we used the abandoned pesticide-contaminated soil as having minimal pesticide exposure to compare with the actively pesticide-contaminated soil to provide a more accurate comparison to the current pesticides spray regime.  Thank you very much. We have introduced the Simpson Index of diversity assessment normally used in ecological study for biodiversity. Already, we included the Sorenson’s similarities assessment in the manuscript to enhance the robustness of the data interpretation. This is found in page 13.  Thank you very much sir. A clear explanation for this discrepancy has been provided in the rewritten discussion. Previous studies have shown that long-term monoculture induce the reduction of beneficial microbes such as Pseudomonas and Bacillus in pesticide-contaminated soils. This may be possible because, cabbage cultivation has been going on well over 30 years on the same parcel of land. Secondly, these bacteria could not have synthesized the carbon source as food and energy. This is found in page 24  Thank you for this valuable and thoughtful comment. The sentence in comment has been simplified and split into more digestible points to infer that *Pseudomonas* *spp*. can survive in extreme environments with or without oxygen and are very important in agriculture, performing several important processes including nitrogen cycling, degradation of aromatic compounds and plant growth promotion. We have also simplified complex points. This is found in page 23  Thank you for this insightful comment. A section has been included to discuss the study's limitations and future potential biases in sampling method and directions. This is found in page 29 |
| **Reviewer 2**   1. **Abstract needs to modify:** The abstract should contain Objectives, Methods/Analysis, Findings, and Novelty/Improvement. In Abstract part (Page 3), Authors should not simply describe soil physical and chemical properties, but should explain innovative and main findings. 2. In Page 6, cabbage should add the Latin name. 3. The introduction is too loosely, written to fail to state explicitly the research questions or hypotheses the study aimed to address. Again, the necessity and innovation of the article should be presented to the introduction. 4. In Page 7, Crops should be supplemented with their Latin names. 5. In Page 8, more details about abandoned pesticide-contaminated and active pesticide-contaminated should be added. 6. Is it appropriate to treat forest soil as non-contaminated soil? 7. The Discussion part was weakest element of the paper. There was no real discussion in terms of the reliability of the results.  - There was also a lack of discussion that really positioned the study and its findings within the context of other similar studies. - References were strategically placed but the academic discussion around these was missing. As a result, it was difficult to see the contribution that this study makes to development and advancement of knowledge in this area.  8. The conclusion part is mostly qualitative description and lacks quantitative data. | Thank you for your comment. We have duly revised the abstract to include the objectives, analytical methods, findings and novelty. The main findings of soil physical and chemical properties have also been described in the abstract. The abstract is found on page 2.  Thank you very much for this comment. The Latin name of cabbage (*Brassica oleracea*) has been added. This is found on page 5.  Thank you for your comments. This comment has been addressed stating clear hypotheses the study aimed to address. This comment has been addressed by rewriting and removing unrelated portions to make the introduction more focused on soil microbiomes  Thank you for the comment. This comment has been addressed in comment 2.  Thank you for this insightful comment. We have actually explained in details what the abandoned pesticide-contaminated and active pesticide-contaminated soils mean. This is found on page 8 and 9  Thank you for this comment. It is appropriate to treat forest soil as non-contaminated because the forest represents a natural state where the functions, structure and composition of soil ecosystems have not been disturbed by recent farm management practices including pesticides application. This was also addressed on page 9  Thank you for your valuable and thoughtful comment. Indeed, we realized there was no real discussion in terms of the reliability of the results. However, we have rewritten the discussion and linked to the results. This is found from page 21 to 28  Thank you for this comment. We have positioned this study and its findings within the context of other previous and similar studies.  Thank you for this comment. This comment has been addressed in comment 7. This comment has been addressed to provide information for a more precise understanding of development and advancement of knowledge in this area.    Thank you for the comment. The conclusion part is made up of both qualitative description and with quantitative data to make it more representative. |
| **Reviewer 3**   1. The study considered three soil samples, so why were the non-contaminated soil samples taken from a pristine forest reserve rather than soil where crops were grown but no pesticides were applied? 2. The article mentions the use of neonicotinoids, bacilli (bio/microbial), and pyrrole pesticides, but does not specify the exact types and concentrations used. It is recommended to provide this information for a more precise understanding of the study's findings. 3. The article only used the Fisher's alpha diversity index, and it is recommended to include additional indices such as the Shannon-Wiener index and Simpson index to provide a more comprehensive assessment of bacterial diversity. 4. The article did not predict bacterial functions, and it is recommended to use databases or functional gene analysis to predict the functions of the bacterial community and explore their potential impact on soil ecosystem functions. 5. The article did not delve into the deeper mechanisms by which pesticides affect bacterial communities. It is recommended to synthesize the results of related studies to analyze the effects of pesticides on bacterial metabolism, growth and reproduction. 6. The overall language of the article is fluent, but some sentences are structurally complex. It is recommended to simplify the language to improve readability. 7. The reference format of the article needs to be checked. | This is an insightful comment, thank you very much. All arable lands in the district have been treated with pesticides because of pest infestation to crops. The forest presented an ideal state where the functions, structure and composition of soil ecosystems have not been disturbed by pesticides  Thank you for this important comment. The used pesticides are grouped under neonicotinoid, bacilli (bio/microbial), avermectin and pyrrole. The active ingredients are acetamiprid, perisrapae granulosis virus+bacillus thuringiensis, emamectin benzoate and chlorfenapyr with trade names as golan 20SL, bypel 1, attack 1.9 EC and klopar respectively. However, the concentration used by the farmers are not known to us. This comment is addressed on page 7.  Thank you for this important comment. We have introduced the Simpson Index of diversity assessment normally used in ecological study for biodiversity for more comprehensive assessment of bacterial diversity. This is found on page 13 and 14 and Table 3.  Thank you for this important comment. We did not predict bacteria functions because, the study objectives were based on effects on composition, abundance and diversity but not on functions  Thank you very much for this comment. We have rewritten the discussion delving into the deeper mechanisms by which pesticides affect bacterial communities. The result has also been synthesized with results of related studies to effectively analyze the effects of pesticides on bacterial metabolism, growth and reproduction.  Thank you for this comment. We have simplified the language to improve readability of the manuscript  Thank you for this comment. We have checked the format of referencing and corrected the wrongs. |
| Thank you once again for the reviewers valuable and thoughtful comments and suggestions. We have made every effort to enhance the manuscript and have implemented several changes which are marked in yellow in the revised paper. However, these modifications do not affect the content or structure of the paper. We sincerely appreciate the diligent work of the Editors and Reviewers and hope that these corrections will meet your kind consideration and approval. | |
