# Supplementary material for: Effects of pesticide application on soil bacteria community structure in a cabbage-based agroecosystem in Ghana
Source: PLoS One. 2025 May 29;20(5):e0323936. doi: 10.1371/journal.pone.0323936 (PMC12121791; doi:10.1371/journal.pone.0323936)
Supplement: Attachment — Submitted filename: Responses to reviewer comments.docx [file pone.0323936.s009.docx]

RESPONSES TO COMMENTS OF REVIEWER

Comment

Abstract: “The applied pesticides also caused significant shift in the pH of the examined soils.” significant shift? What is P value? And more data should be added in Abstract section.

Response

Please, that statement was wrong as there is no data to suggest shifts in pH. Consequently, we have deleted it from the abstract. We have added more data to the abstract.

Comment

In Page 4, “Annually, an estimated two million tons of pesticides are applied on agricultural lands worldwide (7). However, in 2022, the total global pesticide use in agriculture was 3.70 million metric tons (Mt) and a total pesticide trade reaching approximately 7.2 Mt of formulated products amounting to USD 41.1 billion (8).” This sentence is confusing to read and it is suggested that the author rewrite this sentence.

Response

Please, we have deleted this sentence from the manuscript to maintain clarity.

Comment

In Page 12, a low potassium (K) concentration, total potassium or available potassium?

Response

Please, it is total potassium. This has been clarified in the manuscript (please see red font text on page 9)

Comment

In Discussion section, it is suggested to add subheadings for readers' better understanding.

Response

We have provided subheadings in the discussion section.

Comment

The Discussion and Limitation can be combined into Discussion section, and the Conclusion section should be at the end of the manuscript.

Response

We have combined the discussion and the limitations, but because we sub-headed the discussion, we have provided a subheading for the limitations under Discussion.

We have placed the conclusion at the end of the manuscript.
